# Association of Dip in eGFR With Clinical Outcomes in Unilateral Primary Aldosteronism Patients After Adrenalectomy

**DOI:** 10.1210/clinem/dgad709

**Published:** 2023-12-05

**Authors:** Jui-Yi Chen, Kuo-How Huang, Yen-Hung Lin, Jeff S Chueh, Hsien-Yi Wang, Vin-Cent Wu

**Affiliations:** Division of Nephrology, Department of Internal Medicine, Chi-Mei Medical Center, Tainan 71004, Taiwan; Department of Health and Nutrition, Chia Nan University of Pharmacy and Science, Tainan 71710, Taiwan; Department of Urology, College of Medicine, National Taiwan University, Taipei 106319, Taiwan; Department of Urology, National Taiwan University Hospital, Taipei 100225, Taiwan; Department of Internal Medicine, National Taiwan University Hospital, Taipei 100225, Taiwan; Department of Urology, College of Medicine, National Taiwan University, Taipei 106319, Taiwan; Department of Urology, National Taiwan University Hospital, Taipei 100225, Taiwan; Division of Nephrology, Department of Internal Medicine, Chi-Mei Medical Center, Tainan 71004, Taiwan; Department of Sport Management, College of Leisure and Recreation Management, Chia Nan University of Pharmacy and Science, Tainan 71710, Taiwan; Department of Internal Medicine, National Taiwan University Hospital, Taipei 100225, Taiwan

**Keywords:** primary aldosteronism, adrenalectomy, eGFR dip, major adverse kidney event

## Abstract

**Context:**

Primary aldosteronism (PA) leads to kidney function deterioration after treatment, but the effects of the estimated glomerular filtration rate (eGFR) dip following adrenalectomy and its long-term implications are unclear.

**Objective:**

This study aims to examine eGFR dip in patients with unilateral PA (uPA) after adrenalectomy and clarify their long-term prognosis.

**Methods:**

This multicenter prospective population-based cohort study, enrolled patients with uPA who underwent adrenalectomy. Patients were divided into 4 groups based on their eGFR dip ratio. Outcomes investigated included mortality, cardiovascular composite events, and major adverse kidney events (MAKEs).

**Results:**

Among 445 enrolled patients, those with an eGFR dip ratio worse than −30% (n = 74, 16.6%) were older, had higher blood pressure, higher aldosterone concentration, and lower serum potassium levels. During 5.0 ± 3.6 years of follow-up, 2.9% died, 14.6% had cardiovascular composite events, and 17.3% had MAKEs. The group with eGFR dip worse than −30% had a higher risk of MAKEs (*P* < .001), but no significant differences in mortality (*P* = .295) or new-onset cardiovascular composite outcomes (*P* = .373) were found. Multivariate analysis revealed that patients with an eGFR dip ratio worse than −30% were significantly associated with older age (odds ratio [OR], 1.04), preoperative eGFR (OR, 1.02), hypokalemia (OR, 0.45), preoperative systolic blood pressure (OR, 1.03), and plasma aldosterone concentration (OR, 0.99).

**Conclusion:**

Within 5 years post adrenalectomy, 17.3% of patients had reduced kidney function. Notably, individuals with an eGFR dip ratio worse than −30% faced higher MAKE risks, underscoring the need to monitor kidney function in PA patients after surgery.

Primary aldosteronism (PA) is characterized by aldosterone overexcretion, which leads to sodium resorption, volume expansion, increased glomerular filtration, and distal delivery of potassium (1). Consequently, PA has emerged as the most common cause of secondary hypertension (2), chronic kidney disease (CKD) (3), and end-stage kidney disease (ESKD) (4). Furthermore, inappropriate aldosterone excretion has been reported to account for approximately 10% of patients with arterial hypertension (5). Patients with PA have a higher prevalence of CKD compared to those with essential hypertension and a higher risk of worsening kidney function in the long term (6, 7). However, abrupt decline in estimated glomerular filtration rate (eGFR) may be masked in patients with PA after adrenalectomy due to preoperative aldosterone-related hyperfiltration (6). Therefore, it is crucial to better understand the relationship between the degree of acute eGFR change with subsequent eGFR trajectory and long-term outcomes to guide clinical practice.

Maladaptive glomerular hemodynamics is a key factor in the progression of kidney disease, characterized by single nephron hyperfiltration and increased glomerular capillary pressure in response to a reduced number of functional nephrons, independent of the underlying cause (8). Hyperfiltration is frequently observed in PA-related kidney disease and often precedes the development of ESKD (4). Therefore, controlling pathologic hyperfiltration may have therapeutic benefits, even if it leads to an acute decline in eGFR. These findings support the idea that adrenalectomy that cause initial eGFR decreases may have long-term therapeutic benefits for patients with PA-related kidney disease, despite the counterintuitive nature of such an approach.

Therefore, the aim of this study was to investigate the eGFR dip in patients with a diagnosis of unilateral PA (uPA) after adrenalectomy to clarify their prognosis and long-term outcomes; uPA is defined as the overproduction of aldosterone hormone from a single adrenal gland, resulting in excessive sodium retention and potassium excretion.

## Methods

### Patient Selection

**Figure 1.**
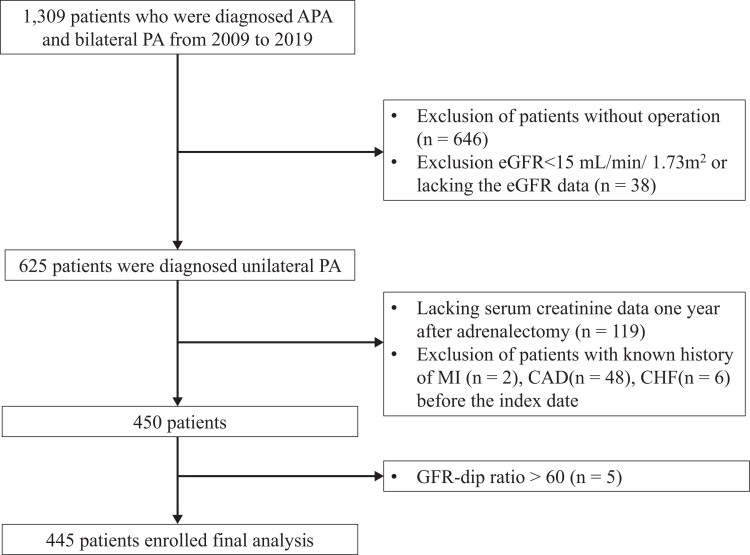
Flow algorithm of patients with unilateral PA after adrenalectomy. Abbreviations: APA, aldosterone-producing adrenal adenoma; CAD, coronary artery disease; CHF, congestive heart failure; eGFR, estimated glomerular filtration rate; MI, myocardial infarction; PA, primary aldosteronism.

In this prospective population-based cohort study, we enrolled 1309 patients diagnosed with aldosterone-producing adrenal adenoma and bilateral PA from January 2009 to June 2019. In order to identify patients with uPA, hypokalemia was corrected and all drugs which may have had an effect on plasma aldosterone or renin concentration were temporarily suspended (9). We then used an aldosterone to renin ratio of > 35 (ng/dL)/ (ng/mL/h) as a screening test (10). For patients with positive screening test results, further confirmation was performed to rule out the possibility of false positive results, including saline infusion test or captopril/losartan suppression test (11). To identify the subtypes of hyperaldosteronism, adrenal computed tomography was performed, and adrenal venous sampling was arranged for lateralization (12, 13). The clinical characteristics and laboratory data were collected at baseline and 6 months after adrenalectomy to assess comorbid conditions and cardiovascular (CV) composite outcomes. Patients with uPA who did not undergo adrenalectomy and those who had a baseline eGFR < 15 mL/min/1.73 m^2^ or nephrotic syndrome were excluded (Fig. 1).

In this study, the index date was set as 6 months after adrenalectomy, and patients who underwent the surgery after June 30, 2019, were excluded. To avoid confounding factors, patients with a history of myocardial infarction, coronary artery disease, or congestive heart failure before the index date were also excluded. The eGFR dip ratio was calculated as the percentage of the difference between 6 months after adrenalectomy and baseline eGFR divided by the baseline eGFR. The association between eGFR dip ratio and major advanced kidney events (MAKEs) was analyzed using a spline plot of log hazard ratio.

The Modification of Diet in Renal Disease (MDRD) equation was used to calculate eGFR as follows: [186 × (serum creatinine)^−1.154^ × (age)^−0.203^ × 0.742 (if female)].

The baseline characteristics including age, sex, body mass index, and comorbidities (Charlson score, diabetes mellitus [DM], hypertension, hyperlipidemia) were recorded. In addition, the medications used before screening including alpha-blockers, beta-blockers, angiotensin-converting enzyme inhibitors/angiotensin II receptor antagonists (ACEis/ARBs), calcium blockers, and diuretics were also recorded. We also measured the plasma aldosterone concentration (PAC), plasma renin activity (PRA), eGFR, potassium, glucose, lipid profile, intact parathyroid hormone (i-PTH), and urine albumin to creatinine ratio during the confirmation stage of PA.

### Outcomes of Interest

The study's primary outcomes were all-cause mortality, MAKE, and new-onset CV composite outcomes. Follow-up of patients was performed according to a standardized protocol, with assessments of eGFR, serum potassium, blood pressure, aldosterone, and renin levels conducted at specific time points, including Day 30, 90, 180, and 360 following adrenalectomy. MAKEs were defined as the occurrence of ESKD, CKD stage progression, or new-onset CKD during follow-up. ESKD was defined as the need for maintenance kidney replacement therapy for at least 90 days. CKD stage progression was identified as an increase in CKD stage after the index date. New-onset CKD was defined as a new diagnosis of CKD stage 3 to 5. The CV composite outcome was defined as a combination of nonfatal myocardial infarction, nonfatal hemorrhagic/ischemic stroke, undergoing coronary artery bypass graft or angiography, atrial fibrillation (Af), congestive heart failure, and CV mortality (14, 15). These events of interests were further validated using the National Health Insurance Research Database (NHIRD) (16), the only national medical copayment program in Taiwan, from January 2010 to December 2019, and ICD9/ICD10 codes (Supplement 1) (17). In order to detect possible fraud in the National Health Insurance (NHI) program, the NHI Administration routinely audits the data and records submitted by healthcare institutions and providers (18). Consequently, the NHIRD can be considered to have high completeness and accuracy.

### Ethical Approval of the Study Protocol

The study was conducted in compliance with the Declaration of Helsinki and was approved by the National Taiwan University Hospital Research Ethics Committee (http://doi.org/10.6084/m9.figshare.21730985). Written informed consent was obtained from all participants prior to their inclusion in the study after receiving comprehensive written information about the study.

## Statistical Analysis

Continuous variables were reported as mean ± SD or median (interquartile range), while categorical variables were presented as number (percentage). Comparisons of continuous variables among 3 groups were made using one-way analysis of variance with post hoc Bonferroni correction or Kruskal–Wallis test, as appropriate. Chi-squared test or Fisher exact test was used to compare categorical variables among different eGFR dip ratio groups. Univariable logistic regression was performed to estimate odds ratios (ORs) and 95% CIs for the associations of baseline factors with the composite outcomes in the PA patients after adrenalectomy. Penalized splines (14, 19) of eGFR against the logarithmic hazard ratio of MAKEs were plotted after adjustments for age, sex, systolic blood pressure, serum potassium, PAC, and PRA to examine potential nonlinear effects of continuous covariates on the outcomes in the regression analysis and identify the appropriate cutoff points to discretize continuous covariates. Factors associated with a decline in eGFR (eg, eGFR dip ratio < −30%) were further calculated with ORs using multivariable logistic regression analysis. Additionally, a Cox proportional hazard model was used to depict the outcomes among different eGFR-dip groups. A Cox proportional hazard model adjusted with aldosterone-producing adrenal adenoma (APA), clinical complete success, partial success, age, sex, serum potassium level, systolic blood pressure, diastolic blood pressure, DM, PAC, PRA, and duration of hypertension was used to depict the outcomes among different eGFR-dip groups. E-values were calculated to evaluate potential confounding due to unmeasured or uncontrolled confounders for the observed association estimates (20). To evaluate for potential confounding, we also assessed the risk of gastrointestinal bleeding as a negative outcome, which is not typically associated with PA. A *P* value < .05 was considered significant. Statistical analyses were performed using STATA/SE version 14.0 for Windows (StataCorp LP, College Station, TX, USA) and R software version 3.4.4 (Free Software Foundation, Inc., Boston, MA, USA).

## Results

### Patient Enrollment

We enrolled 1309 patients who were diagnosed with PA from 2009 to 2019; those who did not undergo adrenalectomy (n = 646), and those with an eGFR < 15 mL/min/1.73 m^2^ or without eGFR data (n = 38) were excluded. In addition, patients with a history of myocardial infarction (n = 2), coronary artery disease (n = 48), and congestive heart failure (n = 6) were excluded, and those without 1 year of postoperative creatinine data after adrenalectomy (n = 119) were also excluded. Among the remaining 450 patients, those with an eGFR dip ratio > 60 (n = 5) were considered to be outliers and were excluded. Finally, 445 patients were included in the analysis (Fig. 1).

### The Characteristics Among Different eGFR-Dip Groups

The mean age of the patients was 50.5 ± 11.1 years, and the mean baseline eGFR was 90.7 ± 27.2 mL/min/1.73 m^2^. The patients were divided into 4 groups: eGFR dip ratio ≥ 0% (n = 60), −15% ≤ eGFR dip ratio < 0% (n = 164), −30% ≤ eGFR dip ratio < −15% (n = 147), and < −30% (n = 74) (Table 1). In general, the patients with an eGFR dip ratio < −30% (74/445, 16.6%) had higher systolic blood pressure, higher aldosterone concentration, and lower serum potassium level. In addition, they had higher baseline eGFR, serum cystatin C, and urine microalbumin to creatinine ratio compared with the other groups.

**Table 1.** Baseline characteristics and clinical information of patients with primary aldosteronism after unilateral adrenalectomy, stratified by eGFR dip ratio

Variables^*f*^	All patients (n = 445)	0 ≤ eGFR dip ratio*^c^* (n = 60)	−15%≤ eGFR dip ratio <0% (n = 164)	−30%≤ eGFR dip ratio < −15% (n = 147)	−30% > eGFR dip ratio (n = 74)	*P* value*^a^*
Age, years	50.5 ± 11.1	49.8 ± 12.5	50.7 ± 10.5	49.9 ± 11.1	52.0 ± 11.4	.57
Sex, male	245 (55.1%)	34 (56.7%)	88 (53.7%)	86 (58.5%)	37 (50.0%)	.65
Body mass index, kg/m^2^	25.3 [22.6-28.0]	25.2 [23.1-27.5]	25.6 [23.2-29.0]	24.6 [22.1-27.34]	25.5 [22.7-27.7]	.22
Medication before screening test						
Number of antihypertensive medications, n [interquartile range]	2 [1-3]	2 [1-3]	2 [1-3]	2 [1-3]	2 [2-3]	.34
alpha-blocker, n (%)	107 (24.0%)	11 (18.3%)	40 (24.4%)	32 (21.8%)	24 (32.4%)	.23
beta-blocker, n (%)	181 (40.7%)	19 (31.7%)	75 (45.7%)	56 (38.1%)	31 (41.9%)	.24
ACEi/ARB, n (%)	187 (42.0%)	19 (31.7%)	68 (41.5%)	63 (42.9%)	37 (50.-%)	.20
Ca-blocker, n (%)	317 (71.2%)	42 (70.0%)	104 (63.4%)	117 (79.6%)	54 (73.0%)	.02
Diuretics, n (%)	44 (9.9%)	7 (11.7%)	16 (9.8%)	11 (7.5%)	10 (13.5%)	.52
Baseline characteristics						
Charlson score	1[0-2]	1[0-2]	1[0-2]	1[0-2]	1[0-2]	.096
DM, n (%)	66 (14.8%)	12 (20.0%)	25 (15.2%)	15 (10.2%)	14 (18.9%)	.19
Hypertension, n (%)	433 (97.3%)	59 (98.3%)	156 (95.1%)	145 (98.6%)	73 (98.7%)	.26
Hyperlipidemia, n (%)	96 (21.6%)	15 (25.0%)	39 (23.8%)	25 (17.0%)	17 (23.0%)	.43
Smoker, n (%)	60 (13.5%)	9 (15.0%)	24 (14.6%)	17 (11.6%)	10 (13.5%)	.86
Hypertension duration (year)	5 [2-10]	4 [2-10]	5 [2-10]	6 [2-10]	5 [2-13]	.42
PAC*^b^* (ng/dL)	45.80 [31.90-71.60]	41.10 [28-66.40]	43.45 [30.29-63.14]	47.71 [30.60-71.60]	60.39 [39.81-90.91]	<.01
PRA*^b^* (ng/mL/h)	0.22 [0.10-0.55]	0.31 [0.13-0.61]	0.20 [0.07-0.54]	0.21 [0.10-0.61]	0.23 [0.10-0.45]	.25
SBP (mmHg)	155.5 ± 21.9	150.9 ± 22.4	153.7 ± 21.3	157.0 ± 22.3	160.4 ± 21.4	.04
DBP (mmHg)	93.8 ± 14.6	92.7 ± 12.7	93.0 ± 15.2	95.2 ± 15.3	93.5 ± 13.0	.52
eGFR (by MDRD, mL/min/1.73 m^2^)	90.7 ± 27.2	74.3 ± 19.0	89.5 ± 22.0	96.1 ± 28.2	95.8 ± 35.1	<.01
Cystatin C	0.85 ± 0.28	0.78 ± 0.24	0.81 ± 0.21	0.84 ± 0.27	0.99 ± 0.41	<.01
Potassium (mEq/L)	3.5 ± 0.6	3.8 ± 0.5	3.7 ± 0.6	3.4 ± 0.6	3.3 ± 0.6	<.01
Glucose (mg/dL)	99.8 ± 19.6	102.5 ± 22.9	100.3 ± 21.4	97.2 ± 16.3	101.7 ± 18.4	.24
Cholesterol (mg/dL)	186.3 ± 37.4	191.7 ± 35.4	187.8 ± 39.5	184.6 ± 34.0	182.3 ± 41.1	.52
Triglyceride (mg/dL)	129.4 ± 85.7	138.6 ± 88.9	138.0 ± 85.9	119.0 ± 90.0	124.7 ± 72.1	.23
HDL (mg/dL)	47.6 ± 13.5	46.7813.8	47.8 ± 14.3	48.4 ± 12.3	46.3 ± 14.2	.72
LDL (mg/dL)	110.3 ± 30.2	113.5 ± 29.6	111.8 ± 30.5	108.1 ± 28.7	109.3 ± 33.0	.65
i-PTH (pg/mL)	72.1 ± 48.0	56.9 ± 24.0	58.6 ± 31.5	84.7 ± 60.7	78.5 ± 46.1	<.01
Urine ACR (mg/g)	0.08 ± 0.31	0.03 ± 0.05	0.04 ± 0.08	0.04 ± 0.07	0.31 ± 0.71	<.01
eGFR dip Ratio*^c^* (%)	−13.4 ± 18.6	18.4 ± 11.2	−5.4 ± 5.7	−22.2 ± 4.5	−39.5 ± 8.8	<.01
postoperative PAC*^g^*	44.6 ± 30.6	52.2 ± 37.8	42.4 ± 29.3	44.1 ± 28.5	45.3 ± 32.2	.454
PAC decline ratio*^g^*	4.5 ± 111.7	15.4 ± 94.9	−1.3 ± 81.1	14.1 ± 152.0	−9.5 ± 82.7	.554
**Outcome**						
Mortality, n (%)	13 (2.9%)	4 (6.7%)	5 (3.1%)	3 (2.0%)	1 (1.4%)	.30
CV Composite outcomes*^d^*, n (%)	65 (14.6%)	13 (21.7%)	24 (14.6%)	18 (12.2%)	10 (13.5%)	.37
MAKE*^e^*	77 (17.3%)	11 (18.3%)	19 (11.6%)	24 (16.3%)	23 (31.1%)	<.01
Follow-up period to CV composite outcomes, years	5.0 ± 3.6	5.4 ± 3.6	5.2 ± 3.7	5.0 ± 3.7	4.0 ± 3.1	.06

Data are expressed as mean ± SD or median (interquartile range).

Abbreviations: ACEi/ARB, angiotensin-converting enzyme inhibitor/angiotensin receptor blockers; ACR, albumin creatinine ratio; Cr, creatinine; CV, cardiovascular; DBP, diastolic blood pressure; DM, diabetes mellitus; eGFR, estimated glomerular filtration rate; HDL, high-density lipoprotein; i-PTH, intact parathyroid hormone; LDL, low-density lipoprotein; MAKE, major adverse kidney events, MDRD, Modification of Diet in Renal Disease; PAC, plasma aldosterone concentration; PRA, plasma renin activity; SBP, systolic blood pressure.

^*a*^the *P* value was calculated using analysis of variance (ANOVA)

^*b*^All antihypertensive medications that would interfere with the RAAS were discontinued before PA confirmation tests.

^*c*^eGFR ratio, the percentage of the difference between 6 months after surgery and baseline eGFR divided by the baseline eGFR, and eGFR was calculated by MDRD methods.

^*d*^Including cardiovascular death, nonfatal myocardial infarction, nonfatal hemorrhage/ischemia stroke, undergoing coronary artery bypass graft or angiography, congestive heart failure, and mortality.

^*e*^MAKE, including the occurrence of end-stage kidney disease, chronic kidney disease stage progression, or new onset of chronic kidney disease during the follow-up period.

^*f*^ Table 1 displays preoperative data, except for the e-GFR dip ratio and clinical outcomes which were measured after the index date, set at 6 months postsurgery.

^*g*^Data were compared by one-way ANOVA with Bonferroni post hoc correction for multiple comparisons.

According to the spline plot (Supplementary Fig. S1**)** (17), the cutoff value of eGFR decline against long-term incident MAKEs for uPA after adrenalectomy was −30%. Therefore, we divided the patients into 2 groups using a 3-dimensional plot: those with an eGFR dip ratio worse than −30% and those with an eGFR dip ratio better than −30%. Comparing these 2 groups, we found that the patients with an eGFR dip ratio worse than −30% were older, had a higher baseline eGFR, and a lower potassium concentration than those with an eGFR dip ratio better than −30% (Supplementary Fig. S2**)** (17).

### Clinical Outcomes Grouped by Different eGFR Dip Ratios

During a mean follow-up period of 5.0 ± 3.6 years, 13 (2.9%) patients died, 65 (14.6%) had CV composite events, and 77 (17.3%) had MAKEs (Table 1). The patients with composite CV outcomes took more diuretics than those without CV composite outcomes (*P* = .01). In addition, the patients with CV composite outcomes had a higher prevalence of type 2 DM (*P* = .1), worse Charlson score (*P* = .01), lower eGFR (*P* < .01), and shorter follow-up period (*P* < .01). There was no significant difference between the 4 eGFR-dip groups in the incidence of CV composite outcomes (eGFR dip ratio ≥ 0%, 21.7%; −15% ≤ eGFR dip ratio < 0%, 14.6%; −30% ≤ eGFR dip ratio < −15%, 12.2%; −30% > eGFR dip ratio, 13.5%; *P* = .37) (Table 2).

**Table 2.** Characteristics of patients with primary hyperaldosteronism after unilateral adrenalectomy grouped by CV composite outcomes

Baseline characteristics	With CV composite outcomes (n = 65)	Without CV composite outcomes (n = 380)	*P* value
Age, years	52.8 ± 12.0	50.1 ± 11.0	.07
Sex, male, n, (%)	32 (49.2%)	168 (44.2%)	.45
Body mass index, kg/m^2^	25.3 [23.7-28.6]	25.2 [22.5-28.0]	.69
Medication before screening test			
Number of antihypertensive medications, n [interquartile range]	2 [2-3]	2 [1-3]	.11
Alpha-blocker, n (%)	19 (29.2%)	88 (23.2%)	.29
Beta-blocker, n (%)	29 (44.6%)	152 (40.0%)	.48
ACEi/ARB, n (%)	29 (44.6%)	158 (41.6%)	.65
Ca-blocker, n (%)	48 (73.9%)	269 (70.8%)	.62
Diuretics, n (%)	12 (18.5%)	32 (8.4%)	.01
Baseline characteristics			
DM, n (%)	14 (21.5%)	52 (13.7%)	.10
Hypertension, n (%)	65 (100.0%)	368 (96.8%)	.23
Hyperlipidemia, n (%)	12 (18.5%)	84 (22.1%)	.51
Smoker, n (%)	10 (15.4%)	50 (13.2%)	.63
Hypertension duration (year)	7[3-14]	5[2-10]	.05
Charlson score	1[1-2]	1[0-2]	.01
PAC*^b^* (ng/dL)	44.9[29.3-61.3]	46.8[32.1-73.0]	.31
PRA*^b^* (ng/mL/h)	0.23[0.08-0.60]	0.22[0.10-0.55]	.94
SBP (mmHg)	158.3 ± 23.4	155.1 ± 21.7	.28
DBP (mmHg)	94.2 ± 15.3	93.7 ± 14.5	.80
eGFR (by MDRD, mL/min/1.73 m^2^)	81.5 ± 26.1	92.3 ± 27.1	<.01
Potassium (mEq/L)	3.4 ± 0.7	3.6 ± 0.6	.09
Glucose (mg/dL)	99.5 ± 21.1	99.8 ± 19.4	.9
Cholesterol (mg/dL)	186.5 ± 50.8	186.2 ± 34.9	.96
Triglyceride (mg/dL)	137.5 ± 88.2	128.1 ± 85.3	.45
HDL (mg/dL)	44.2 ± 13.7	48.1 ± 13.4	.05
LDL (mg/dL)	102.5 ± 32.9	111.5 ± 29.7	.05
i-PTH (pg/mL)	74.5 ± 36.7	71.9 ± 49.0	.81
Urine ACR (mg/g)	0.1 ± 0.3	0.1 ± 0.3	.58
eGFR Ratio*^a^* (%)	−10.8 ± 20.3	−13.9 ± 18.3	.22
eGFR group			.37
ratio > 0%	13 (20.0%)	47 (12.4%)	
−15%≤ eGFR dip ratio <0%	24 (37.0%)	140 (36.8%)	
−30%≤ eGFR dip ratio < −15%	18 (27.7%)	129 (34.0%)	
−30% > eGFR dip ratio	10 (15.4%)	64 (16.8%)	
Follow-up period (year)	3.3 ± 3.2	5.3 ± 3.6	<.01

Data are expressed as mean ± SD or median (interquartile range).

eGFR was calculated by MDRD.

Abbreviations: ACEi/ARB, angiotensin-converting enzyme inhibitor/angiotensin receptor blockers; CV, cardiovascular; DM, diabetes mellitus; eGFR, estimated glomerular filtration rate; HDL, high-density lipoprotein; i-PTH, intact parathyroid hormone; LDL, low-density lipoprotein; MDRD, Modification of Diet in Renal Disease; PAC, plasma aldosterone concentration; PRA, plasma renin activity.

^*a*^eGFR ratio, the percentage of the difference between 6 months after surgery and baseline eGFR, divided by the baseline eGFR.

^*b*^All antihypertensive medications that would interfere with the RAAS were discontinued before PA confirmation tests.

The crude incidence rates of CV composite outcomes were 3993, 2791, 2471, and 3393 per 100 000 person-years for the 4 eGFR-ratio-dip groups, respectively (Supplementary Table S1) (17). Baseline hypokalemia was found to be associated with CV composite outcomes (potassium level, hazard ratio [HR], 0.58, 95% CI [0.36-0.95], *P* = .03) (Table 3). However, an eGFR dip ratio worse than −30% was not significantly associated with the risk of CV composite outcomes in adjusted Cox proportional analysis (*P* = .58). Multivariable logistic regression analysis was performed to evaluate the characteristics of the patients with an eGFR dip ratio better than −30%, which revealed that the significant factors were younger age (OR, 0.96, 95% CI [0.93-1.00]); preoperative eGFR (OR, 0.98, 95% CI [0.97-0.99]); hyperkalemia (potassium level, OR, 2.22, 95% CI [1.39-3.54]); preoperative systolic blood pressure (OR, 0.97, 95% CI [0.96-0.99]); and PAC (OR, 0.99, 95% CI [0.98-1.00]) (Table 4). Older age, male, higher systolic blood pressure (SBP), DM, PAC, and duration of hypertension were found to be associated with MAKEs (age, HR, 1.04, 95% CI [1.02-1.07], *P* = .01; male, HR, 3.01, 95% CI [1.87-4.83], *P* = .01; SBP, HR, 1.03, 95% CI [1.02-1.05], *P* = .01; DM, HR, 1.90, 95% CI [1.16-3.10], *P* = .01; PAC, HR, 1.01, 95% CI [1.00-1.01], *P* = .01; duration of hypertension, HR, 1.04, 95% CI [1.01-1.07], *P* = .02) (Supplementary Table S2) (17).

**Table 3.** Baseline factors associated with cardiovascular composite outcomes for patients with unilateral primary aldosteronism after adrenalectomy

Parameter	Hazard ratio (95% CI)	*P* value
Clinical complete success*^a^*	0.50 (0.23-1.07)	.07
Partial success*^b^*	0.73 (0.34-1.54)	.40
age ≥ 63 years	1.15 (0.52-2.56)	0.73
Male	1.38 (0.78-2.45)	.27
K (per 1 mmol/L)	0.58 (0.36-0.95)	.03
SBP (≥160 mmHg)	1.28 (0.68-2.43)	.45
DBP (per 1 mmHg)	1.00 (0.97-1.02)	.68
DM (yes)	2.07 (1.03-4.19)	0.04
PAC (ng/dL)*^c^*	0.99 (0.98-1.00)	.06
PRA (ng/dL) *^c^*	0.99 (0.87-1.11)	.81
HTN_duration (years)	1.03 (0.99-1.07)	.18
eGFR dip ratio > 0 (reference)		
−15% ≤ eGFR dip ratio <0%	0.91 (0.39-2.12)	.82
−30% ≤ eGFR dip ratio < −15%	0.86 (0.36-2.09)	.74
−30% > eGFR dip ratio	0.74 (0.26-2.14)	.58

Abbreviations: DBP, diastolic blood pressure; DM, diabetes mellitus; eGFR, estimated glomerular filtration rate; HTN, hypertension; K, potassium; PAC, plasma aldosterone concentration; PRA, plasma renin activity; SBP, systolic blood pressure.

^*a*^Normal blood pressure without the aid of antihypertensive medication

^*b*^The same blood pressure as before surgery with less antihypertensive medication or a reduction in blood pressure with either the same amount or less antihypertensive medication.

^*c*^All antihypertensive medications that would interfere with the RAAS were discontinued before PA confirmation tests.

**Table 4.** Multivariate logistic regression analysis showing the patient characteristics associated with an eGFR dip ratio better than −30%

Variables	Odds ratio (95% CI)	*P* value
Age, years	0.96 (0.93-1.00)	.02
Male	0.59 (0.33-1.04)	.07
eGFR (by MDRD)	0.98 (0.97-0.99)	.01
**Potassium** (per 1 mmol/L)	2.22 (1.39-3.54)	.01
SBP (per 1 mmHg)	0.97 (0.96-0.99)	.01
DBP (per 1 mmHg)	1.03 (1.00-1.06)	0.06
DM	0.54 (0.26-1.11)	.09
PAC (ng/dL)***^a^***	0.99 (0.98-1.00)	.01
PRA (ng/dL)***^a^***	1.06 (0.84-1.34)	.64
Hypertension duration (years)	0.99 (0.94-1.03)	.51

Abbreviations: DBP, diastolic blood pressure; DM, diabetes mellitus; eGFR, estimated glomerular filtration rate; MDRD, Modification of Diet in Renal Disease; PAC, plasma aldosterone concentration; PRA, plasma renin activity; SBP, systolic blood pressure.

^*a*^All antihypertensive medications that would interfere with the RAAS were discontinued before PA confirmation tests.

**Figure 2.**
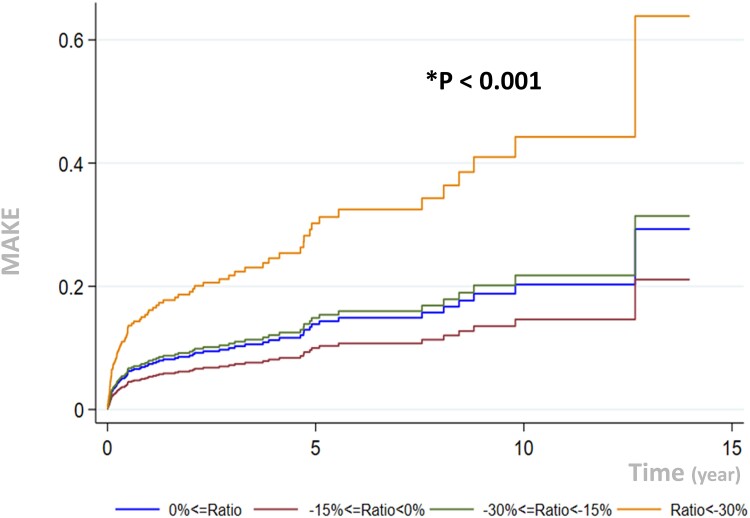
Curve of Cox proportional hazards depicting the major adverse kidney events outcome for patients with unilateral primary aldosteronism after adrenalectomy, grouped according to different eGFR dip ratio values. (*P* < .001, group of ratio < −30% compared with ratio ≥ 0%). The eGFR dip ratio is defined as the percentage of the difference between 6 months after adrenalectomy and baseline eGFR divided by the baseline eGFR. A Cox proportional hazard model adjusted with aldosterone-producing adrenal adenoma (APA), clinical complete success, partial success, age, sex, serum potassium level, systolic blood pressure, diastolic blood pressure, DM, PAC, PRA, duration of hypertension was used to depict the outcomes among different eGFR-dip groups. Abbreviations: eGFR, estimated glomerular filtration rate; PA, primary aldosteronism.

Cox proportional hazards analysis showed that an eGFR dip ratio worse than −30% was significantly associated with a higher risk of incident MAKEs (HR, 2.18, 95% CI [1.04-4.57], *P* < .001) (Fig. 2). The E-value was 3.61, demonstrating moderate unmeasured confounding (20).

In negative outcome analysis, the patients with an eGFR dip ratio worse than −30% had a similar risk of gastrointestinal bleeding compared to those with an eGFR dip ratio ≥ 0% (*P* = .58) after adjusting for the same covariables as in the main analysis (Supplementary Table S3) (17).

## Discussion

Our study demonstrated that a substantial proportion of patients with uPA develop a significant decline in eGFR after adrenalectomy. Specifically, 16.6% of the patients with uPA had an eGFR dip ratio worse than −30%. These patients were characterized by higher baseline blood pressure, serum aldosterone concentration, hypokalemia, and eGFR. Importantly, we found that patients with an eGFR dip ratio worse than −30% had the highest risk of developing long-term incident MAKEs after a mean 5 years of follow-up. However, there was no significant difference in CV composite outcomes among the different eGFR dip ratio groups. These findings suggest that careful monitoring of renal function is warranted in patients with uPA after adrenalectomy, particularly in those with an eGFR dip ratio worse than −30% (Supplement, graph abstract) (17).

### The Characteristics of the Patients With an eGFR Dip Ratio Better Than −30%

Our results showed that a younger age, higher baseline eGFR, lower potassium level, higher preoperative PAC, and higher SBP were associated with a postoperative eGFR dip ratio better than −30%. A higher baseline eGFR implies relative kidney hyperfiltration due to excessive aldosterone in PA patients (21), and the kidney damage may be masked by increased kidney perfusion (22). After adrenalectomy, a significant decrease in eGFR has been demonstrated in several studies (23). On the other hand, Ribstein et al reported that preoperative plasma potassium was a significant determinant of the change in GFR (24), and Iwakura et al also reported that preoperative hypokalemia was independently associated with a severe decrease in GFR after an intervention, including adrenalectomy (23). In line with our results, patients with preoperative hypokalemia and a long vintage of hypertension have also been reported to have a greater risk of a decrease in eGFR after adrenalectomy (25), and chronic hypokalemia has been associated with tubulointerstitial damage including vacuolization of epithelial tubular cells and interstitial fibrosis (26), which was compatible with our result, patients with an eGFR dip ratio worse than −30% had higher risk of hypokalemia.

### Outcomes of the Patients With an eGFR Dip Ratio Worse Than −30%

In the general population, decreased eGFR is independently associated with increased CV disease and mortality (27, 28). A systematic review of 14 randomized trials of interventions reported that when the eGFR had dropped to less than −30%, further decreases in eGFR were not attributed to worse outcomes (29). Several interventions affecting the risk of renal function decline have been associated with “permissive” impairment of kidney function, without an appreciable effect on CKD or mortality (29). While these may be the result of permissive acute kidney injury (AKI), there's limited exploration in the literature about populations with a greater GFR dip. Several studies have reported an association between kidney parenchymal injury with a decrease in eGFR of −30%, this was actually a reflection of hemodynamic change (30-32). For example, sodium–glucose cotransporter 2 inhibitors have shown a kidney-protective effect in patients with permanent tubular damage, however in patients with an eGFR dip ratio worse than −30%, they have also been associated with a deterioration in kidney function (33). The observed coexistence of eGFR declines and long-term clinical benefits may seem paradoxical, but it has been previously described in the field of nephrology. Similar phenomena have been observed with renin-angiotensin-aldosterone system inhibitors (34, 35). For example, ADVANCE Trial, patients with acute increases in serum creatinine after starting ACEi therapy were categorized into 4 groups based on the percentage of increase (<10%, 10% to 19%, 20% to 29%, and ≥30%). The study found that acute increases in serum creatinine after initiating ACEi were associated with short-term risks of major adverse cardiac events, new or worsening nephropathy, and all-cause mortality. However, continuing perindopril treatment reduced the long-term risk of major clinical outcomes, regardless of the acute increase in serum creatinine, compared to patients who stopped the drug (36).

Intensive blood pressure control was shown to result in a rapid decline in eGFR more than 30% compared with the control group in the SPRINT study (30), but not increases in levels of urinary biomarkers of tubular cell damage (37). This reflects hemodynamic changes rather than intrinsic kidney injury despite the loss of GFR. On the basis of these and our results, the feasibility and validity of using a decline in GFR of more than 30% as an endpoint is still under debate, and different trials may yield different outcomes (38). In this study, no significant difference was found in the CV composite outcomes between the different eGFR dip ratio groups. This raises the possibility that the negative effects of aldosterone-related CV events, even in the presence of mild kidney injury, may outweigh or be similar to the impact of severe kidney injury.

Several factors could contribute to the phenomenon for the lack of a significant association between eGFR dip and CV outcomes. First, PA involves unique pathophysiological pathways that can influence both kidney and cardiovascular outcomes. While aldosterone excess can lead to fluid retention and hypertension, which are risk factors for cardiovascular disease, it can also directly affect the kidneys by promoting fibrosis and inflammation. Second, the time frame of observation may be limited to follow-up cardiovascular events. Third, patients who underwent adrenalectomy may receive interventions specifically aimed at reducing the risk of cardiovascular events, such as medications for blood pressure or cholesterol management. Fourth, the characteristics of patients with PA, including their baseline cardiovascular risk and the severity of their hyperaldosteronism, could also modulate the relationship between eGFR dip and cardiovascular outcomes (39). Therefore, the degree of GFR decline alone may not be the only factor associated with composite CV outcomes. On the other hand, interventions that increase eGFR have not been shown to improve outcomes. A striking example of this is bardoxolone, where an improvement in eGFR did not result in a reduction in the risk of ESKD or death from cardiovascular causes (40). Taken together, our findings suggest the concept of “permissive hypercreatinemia,” which emphasizes the importance of accepting a modest decline in eGFR as a necessary trade-off for initiating and maintaining treatment that provide significant long-term benefits for patients (41). On the other hand, patients with an eGFR dip ratio worse than −30% had a significantly higher risk of MAKEs during the follow-up period. This suggests that a decline in eGFR of more than 30% may result from permanent tubular damage rather than hemodynamic changes. Hence, careful monitoring of kidney function and modification of early risk factors are essential in the management of PA patients after adrenalectomy, especially those with a baseline eGFR dip ratio worse than −30%.

Compared to Kobayashi et al's study (21), they found those had PA, after treatment with a mineralocorticoid receptor antagonist and adrenalectomy, showing a correlation between the acute decrease in eGFR and the long-term decline in eGFR in the mineralocorticoid receptor antagonist group. However, no such correlation was observed with adrenalectomy. This finding differs from our study. We believe this difference may be due to the method used to categorize the degree of GFR decline. In our study, we utilized GFR dip, which is calculated as the percentage difference between eGFR at 6 months after surgery and the baseline eGFR, divided by the baseline eGFR. By considering the baseline eGFR, we accounted for its impact. Additionally, in contrast to the study conducted by Kobayashi et al, our study had a specific focus on examining the correlation between various degrees of GFR dip following adrenalectomy and outcomes such as mortality, cardiovascular composite events, and MAKEs.

### Strengths and Limitations

This study has several strengths. Firstly, it is the first study to clearly identify the degree of kidney function decline (eGFR dip ratio worse than −30%) and its long-term outcomes in PA patients after adrenalectomy. Our study is novel in that it is the first to report the association between eGFR dip after adrenalectomy and mortality or CV events in PA patients. Previous studies have mainly focused on the association between eGFR dip and renal outcomes, such as ESRD or CKD (22, 42). For example, we found that adrenalectomy improved the long-term risk of ESRD and mortality of PA patients compared with those treated with mineralocorticoid receptor antagonists, and that patients with an eGFR dip ratio worse than 30% had a higher risk of ESRD than those with an eGFR dip ratio better than 30%. However, they did not report the association between eGFR dip and mortality or CV events separately. Similarly, Srougi et al (42) reported that predictors of complication after adrenalectomy included tumor size, malignancy, and preoperative eGFR, but they did not analyze the association between eGFR dip and long-term outcomes. Therefore, our study provides new insights into the prognostic value of eGFR dip after adrenalectomy for PA patients. Clinicians should maintain heightened awareness of MAKE risk factors in uPA patients, even if their preoperative GFR falls within an acceptable range. Secondly, we have shown that an eGFR dip ratio worse than −30% is associated with a higher risk of incident MAKEs, while there is no significant difference in CV composite outcomes among the 4 eGFR dip ratio groups after adrenalectomy. Additionally, the research provides valuable information by establishing a clear lower limit for postoperative GFR in uPA patients, offering clinical teams a reference point for intervention (43). Thirdly, the population in Taiwan is covered by the NHI program, which may have reduced ascertainment bias in the outcomes after long-term follow-up. Fourthly, although the associations found in this observational study may be biased by unmeasured confounding and do not imply a causal relationship, the E-value of an eGFR dip ratio worse than −30% for the risk of composite MAKEs was greater than known risk factors in patients with PA (sSupplementary Table S2) (17), and the risk of incident gastrointestinal bleeding was similar between groups. Thus, it is less likely that unmeasured bias could explain the observed effects. The specific actions we had taken for the potential confounding factors in our analysis were illustrated in the supplement 2 (17).

The study has some limitations that should be considered. Firstly, the MDRD equation was used to calculate eGFR based on serum creatinine levels, which may not be accurate for individuals without CKD or those with kidney hyperfiltration. Secondly, PA-induced hyperfiltration may obscure other underlying causes of GFR decline, necessitating a comprehensive evaluation of contributing factors. Thirdly, due to the observational nature of the study, definitive conclusions on treatment advantages cannot be drawn as there was no randomization. Fourthly, kidney function was not checked repeatedly at each time point, which could have led to information bias. Fifthly, the findings may not be generalizable to other populations or ethnic groups and require confirmation with larger sample sizes. Sixthly, the eGFR dip ratio worse than −30% and the risk of MAKE represent an association, not a causality relationship. Seventhly, our primary research focus did not center around the investigation of clinical outcomes based on pathological classification. Finally, 13% of patients did not have follow-up creatinine data after adrenalectomy, and patients with an eGFR dip were more likely to receive frequent follow-up, which could have led to cointervention and proficiency biases.

## Conclusion

In summary, this study highlights that a significant proportion of patients with uPA may experience a decline in kidney function after adrenalectomy, with one sixth having an eGFR dip ratio worse than—−30%. Younger patients with higher baseline eGFR, lower potassium level, higher preoperative PAC, and higher SBP are more likely to experience a significant decline in kidney function after adrenalectomy. Patients with an eGFR dip ratio worse than—−30% are at higher risk for adverse kidney events and should be closely monitored. However, no significant differences were observed in CV composite outcomes and mortality between the different eGFR dip ratio groups. These findings underscore the importance of comprehensive evaluation of kidney function and risk factor modification in the management of patients with uPA after adrenalectomy.

## Data Availability

The data that support the findings of this study are available from the corresponding author upon reasonable request.

## Abbreviations

ACEi: angiotensin-converting enzyme inhibitor
ARB: angiotensin II receptor antagonist
CKD: chronic kidney disease
CV: cardiovascular
DM: diabetes mellitus
eGFR: estimated glomerular filtration rate
ESKD: end-stage kidney disease
HR: hazard ratio
i-PTH: intact parathyroid hormone
MAKE: major adverse kidney event
MDRD: Modification of Diet in Renal Disease
NHI: National Health Insurance
NHIRD: National Health Insurance Research Database
OR: odds ratio
PA: primary aldosteronism
PAC: plasma aldosterone concentration
PRA: plasma renin activity
uPA: unilateral primary aldosteronism
